# Supplementary material for: Single-cell fluidic force microscopy reveals stress-dependent molecular interactions in yeast mating
Source: Commun Biol. 2021 Jan 4;4:33. doi: 10.1038/s42003-020-01498-9 (PMC7782832; doi:10.1038/s42003-020-01498-9)
Supplement: Supplementary file 2 — Supplementary Information [file 42003_2020_1498_MOESM2_ESM.pdf]

Supplementary Information for

Single-cell fluidic force microscopy reveals stress-dependent molecular interactions in yeast mating

Marion Mathelié-Guinlet[1], Felipe Viela[1], Jérôme Dehullu[1], Sviatlana Filimonava[2], Jason M. Rauceo[2], Peter N. Lipke[3], and Yves F. Dufrêne[1]

[1]Louvain Institute of Biomolecular Science and Technology, UCLouvain, Croix du Sud, 4-5, bte L7.07.07, B-1348 Louvain-la-Neuve, Belgium

[2] Department of Sciences, John Jay College of the City University of New York, New York, NY 10019, USA

[3]Biology Department, Brooklyn College of the City University of New York, 2900 Bedford Avenue, Brooklyn, NY 11210 USA

[*]Corresponding authors:

Yves Dufrêne: yves.dufrene@uclouvain.be

Peter Lipke: PLipke@brooklyn.cuny.edu

This PDF file includes supplementary figures 1 to 4.

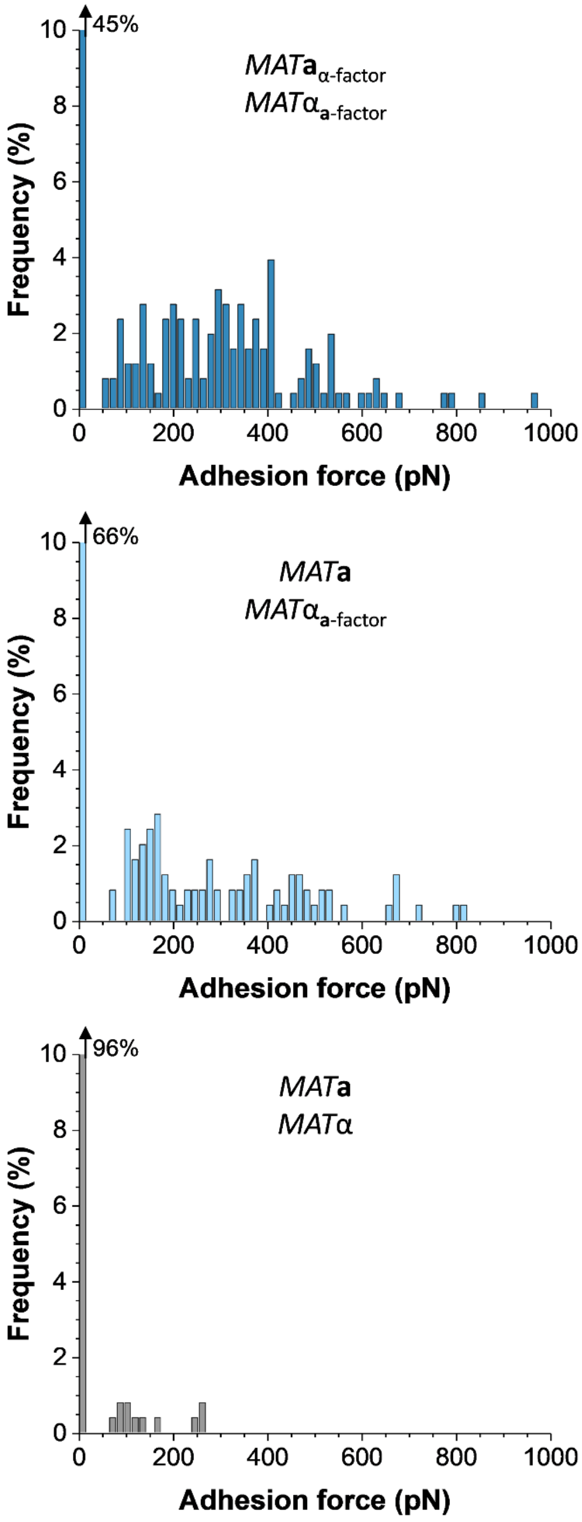

Supplementary Figure 1. Pheromone induction is crucial to yeast agglutination. Maximum adhesion force histograms obtained by recording force-distance curves in PBS between *MAT***a** and *MAT*α cells under diverse pheromones treatment: (i) both mating types are treated with the opposite mating pheromones (*MAT***a**$_{\alpha\text{-factor}}$ and *MAT*α$_{\mathbf{a}\text{-factor}}$), (ii) only *MAT*α cells are treated with *MAT***a** pheromones (*MAT***a** and *MAT*α$_{\mathbf{a}\text{-factor}}$) and (iii) none cells were pre-treated with pheromones (*MAT***a** and *MAT*α). Percentage on the top left corner stands for the non-adhesive events.

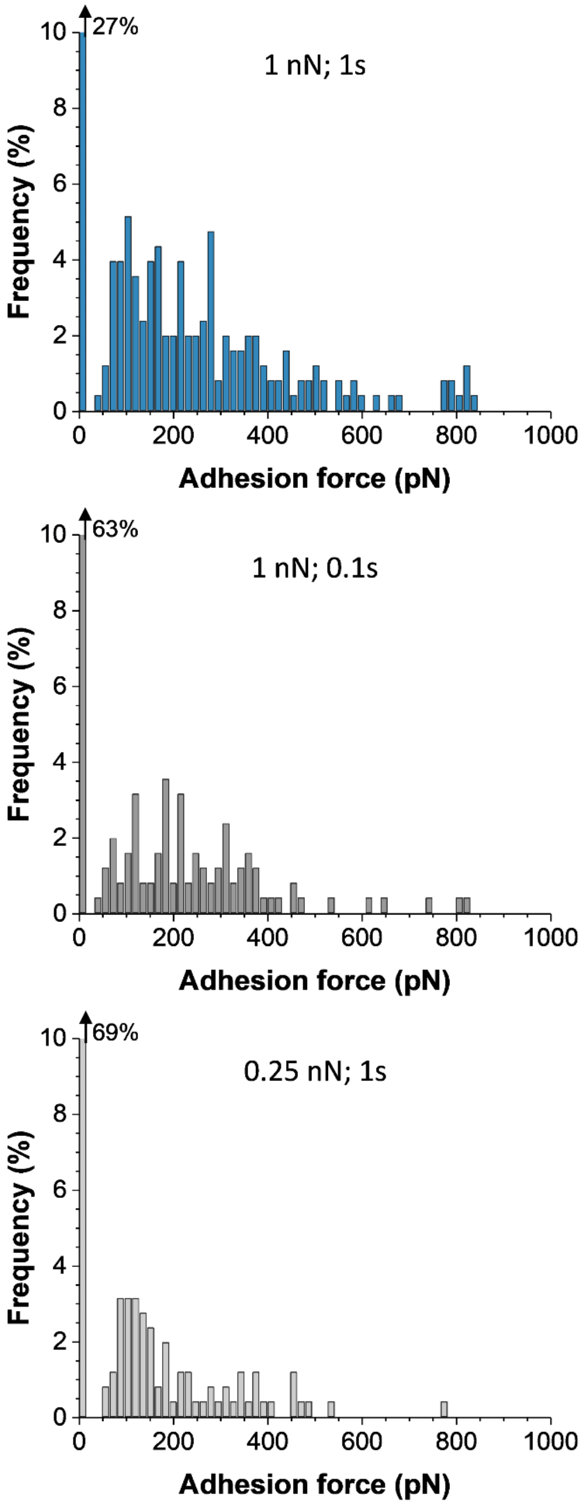

Supplementary Figure 2. Single a- and α-agglutinins interaction is ~100 pN strong. Maximum adhesion force histograms obtained by recording force-distance curves in PBS between *MAT***a** and *MAT*α cells under different applied forces (1 nN or 0.25 nN) and with different probing times (1 s or 0.1 s). Percentage on the top left corner stands for the non-adhesive events.

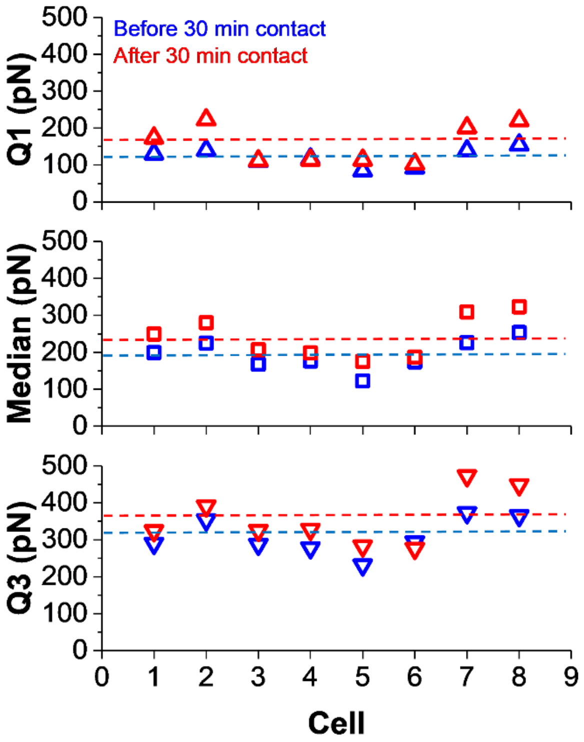

Supplementary Figure 3. Prolonged cell-cell contact increases agglutination forces. Scatter plots of the adhesion forces reached at diverse quartiles (Q1: 25 %-, median: 50 %- and Q3: 75 %- of the adhesive population) when recording force-distance curves between independent *MAT***a** and *MAT*α cell-pairs, before or after 30 min cell-cell contact. Dashed lines are a guide for the eye.

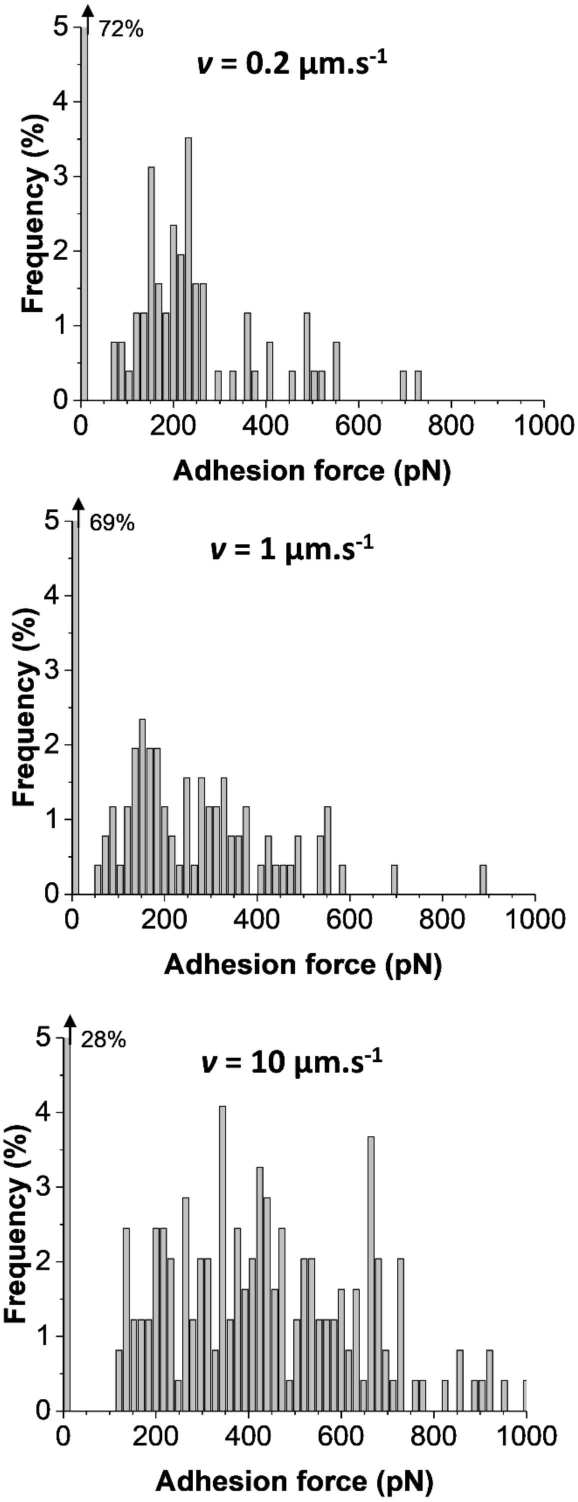

Supplementary Figure 4. Agglutination interactions are enhanced by mechanical stress. Maximum adhesion force histograms obtained by recording force-distance curves in PBS between *MAT***a** and *MAT*α cells, with an applied force of 1 nN, a probing time of 1 s, and at varied retraction speed *v.*
