# Supplementary material for: Single-cell fluidic force microscopy reveals stress-dependent molecular interactions in yeast mating
Source: Commun Biol. 2021 Jan 4;4:33. doi: 10.1038/s42003-020-01498-9 (PMC7782832; doi:10.1038/s42003-020-01498-9)
Supplement: Supplementary file 3 — Description of Additional Supplementary Files [file 42003_2020_1498_MOESM3_ESM.pdf]

Description of Additional Supplementary items

File name: Source data

Description: The Source data underlying main figures 1 to 6 and supplementary figures 1 to 4
